# Proteomic-based identification of APCS as candidate protein for diagnosis of patients exhibiting anti-tubercular drug induced liver injury

**DOI:** 10.1038/s41598-023-35930-x

**Published:** 2023-06-22

**Authors:** Bhavneet Kaur, Ravi Dixit, Shikha Bakshi, Monidipa Konar, Saroj K. Sinha, Ajay Kumar Duseja, Sadhna Sharma

**Affiliations:** 1 Department of Biochemistry, Postgraduate Institute of Medical Education and Research, Chandigarh, 160012 India; 2 Department of Gastroenterology, Postgraduate Institute of Medical Education and Research, Chandigarh, 160012 India; 3 Department of Hepatology, Postgraduate Institute of Medical Education and Research, Chandigarh, 160012 India

**Keywords:** Biochemistry, Molecular biology, Systems biology, Biomarkers, Medical research

## Abstract

Traditional markers evaluate anti-tubercular drug-induced liver injury (AT-DILI). However, these markers have certain limitations and studies are in progress to characterize AT-DILI at an early stage. In the present study, 40 patients were categorized and equally distributed into healthy controls, newly diagnosed tuberculosis (TB), TB without hepatotoxicity and TB with hepatotoxicity groups based on their conventional liver function tests. Relative protein quantification was performed on depleted pooled serum samples of each representative group by LC–MS/MS, and validation of shortlisted protein was done by ELISA. Levels of all analysed biochemical parameters showed a statistical increment in the hepatotoxicity group compared to the other three groups, representing AT-DILI. Comparative proteomic analysis between TB with hepatotoxicity versus TB without hepatotoxicity groups highlighted 24 significant differentially expressed proteins, including PROS1, KNG1, CFH, LCAT, APCS and ADIPOQ. Identified proteins were involved in complement activation, triglyceride-rich lipoprotein particle remodelling and pathways comprising complement, coagulation cascades and cholesterol metabolism. Based on functional relevance, the serum amyloid P component (APCS) was shortlisted for validation, and it showed a similar trend as observed in the discovery phase with 100% sensitivity and 87% specificity; however, findings need exploration in larger cohorts.

## Introduction

India is considered as one of the high tuberculosis (TB) burden countries and accounts for two-third of the total global cases^1^. The currently followed drug regimen for TB treatment is Isoniazid (INH), Rifampicin (RIF), Pyrazinamide (PZA), Ethambutol (EMB) daily for 2 months (initial phase) followed by INH and RIF daily for 4 months (continuous phase)^2^. Although the current anti-TB drug therapy is effective, still it is associated with various side effects predominantly hepatotoxicity and peripheral neuritis.

Anti-tubercular drug-induced liver injury (AT-DILI) is one of the major reason for the development of hepatotoxicity which can lead to treatment failure among TB patients^3^. Frequency of AT-DILI varies from 2 to 39% among different countries^4^. Some cross-sectional and retrospective studies have reported 7.9% incidence and 9.48% prevalence of hepatotoxicity among TB patients on anti-tuberculosis therapy^5,6^. INH, RIF and PZA are the main causative agents for the development of hepatotoxicity in TB patients^7–9^. Additionally, case control studies have shown that TB patients with older age, associated comorbid conditions, chronic alcoholics, suffering from chronic liver diseases and having lower body mass are more susceptible to develop drug-induced hepatotoxicity^6,10^. Anti-TB drug-induced hepatotoxicity can lead to irreversible liver failure that may require liver transplantation and, if not recognized, can be fatal^11^. Current diagnosis involves analysis of various liver function parameters like alanine aminotransferase (ALT), aspartate aminotransferase (AST) and total bilirubin (TBIL). However, AT-DILI lacks differential diagnosis and often is characterized similar to other forms of DILI as AST and ALT levels possess poor specificity for the liver.

The advancement in omics-based technologies, including genomics, proteomics and metabolomics can serve as an important tool to unravel the mechanism of DILI^12^. Mass spectrometry-based high throughput technique is the best approach to profile global protein expression, which can be employed in complex biological samples including serum to quantify proteins^13^. The efficiency to identify novel biomarkers from clinical specimens has dramatically improved due to quantitative proteomics approaches^14^. Serum biomarkers, including glutamate dehydrogenase, keratin 18, glutathione S-transferase and many others, have recently been documented as potential biomarkers to characterize DILI but are yet in their infancy and need further exploration^15^.

Hence, in the present study, we hypothesized that the proteomics approach would identify potential proteins with high discriminative ability in TB patients with and without hepatotoxicity. Further validation of differentially expressed proteins in serum of TB patients on anti-tubercular drugs may lead to the identification of protein signatures which may  play a crucial role in development of AT-DILI and could be plausible therapeutic targets.

## Results

### Patient recruitment and study groups

Study subjects were recruited based on the inclusion and exclusion criteria and were categorized into different groups (Table 1) based on various liver function parameters. The demographic profile of patients along with biochemical parameters is summarized in Tables 2 and 3 respectively. The mean age of hepatotoxic patients was relatively higher than the other three groups; however, the findings were non significant. The TB patients irrespective of drug treatment or hepatotoxicty, were found to have significant low BMI as compared to healthy control. Patients recruited in the study were not having any other associated comorbidities, like diabetes, HIV or other chronic liver disease. A significant increase was seen in the serum levels of alanine aminotransferase (ALT), aspartate aminotransferase (AST), total bilirubin (TBIL), alkaline phosphatase (ALP) and gamma-glutamyl transferase (GGT) (Table 3) in Group 4 patients as compared to Groups 1, 2 and 3 indicating hepatotoxicity in Group 4 patients.

**Table 1:** Description of study groups.

Groups	Description
Group 1: Healthy control	The subjects had no symptoms of TB and were free from any liver disease
Group 2: Newly diagnosed TB subjects	Diagnosed TB patients not yet treated with anti-TB drugs
Group 3: TB subjects without hepatotoxicity	TB patients were taking anti-TB drugs but were not diagnosed with DILI
Group 4: TB subjects with hepatotoxicity	TB patients had been diagnosed with DILI after taking anti-TB drugs (ALT or AST ≥ three-fold rise)

**Table 2:** Demographic details of study subjects enrolled in different groups.

Groups	Age (years)		Sex			BMI (kg/m^2^)	
	Mean ± SD	*p*-value	Male	Female	*p*-value	Mean ± SD	*p*-value
Group 1: Healthy	25.12 ± 2.8	0.120^ ns^	70% (7)	30% (3)	0.361^ ns^	25 ± 1.23	< 0.0001****
Group 2: Newly diagnosed TB subjects	26.31 ± 6.34		70% (7)	30% (3)		18.23 ± 2.75	
Group 3: TB subjects without hepatotoxicity	27.35 ± 5.28		40% (6)	60% (9)		17.10 ± 3.59	
Group 4: TB subjects with hepatotoxicity	31.28 ± 9.51		60% (9)	40% (6)		17.00 ± 1.25	

Parenthesis indicate number of subjects. *p*-value calculated using chi-square test and one-way ANOVA with post-hoc Dunnett’s test. Mutiple group comparisons were made keeping healthy group as control and other groups as test.

**Table 3:** Serum biochemical analysis of included study subjects.

Variable	Group 1 (n = 10) G1	Group 2 (n = 10) G2	Group 3 (n = 15) G3	Group 4 (n = 15) G4	Adjusted *p*-value G1 versus G4	Adjusted *p*-value G2 versus G4	Adjusted *p*-value G3 versus G4
ALT (U/L)	13.34 ± 1.82	8.12 ± 1.44	15.98 ± 2.54	130.90 ± 23.39	0.0027**	< 0.0001****	0.0099**
AST (U/L)	21.89 ± 2.37	18.22 ± 1.69	18.80 ± 2.51	241.00 ± 86.94	0.0062**	0.0004***	0.0002***
ALP (U/L)	75.90 ± 7.71	80.20 ± 7.50	63.80 ± 5.07	190.00 ± 36.95	0.0071**	0.0303*	0.0003***
T.BIL. (mg/dL)	0.29 ± 0.06	0.25 ± 0.06	1.93 ± 1.68	5.42 ± 2.76	0.0145*	0.0035**	0.0276*
GGT (U/L)	24.00 ± 8.55	24.40 ± 6.15	23.90 ± 3.55	70.50 ± 6.70	0.0020**	0.0040**	0.0066**
Total Protein (g/dL)	6.92 ± 0.21	7.52 ± 0.33	6.59 ± 0.44	7.35 ± 0.36	> 0.9999	> 0.9999	> 0.9999

Values are Mean ± SEM.

G1 (Healthy), G2 (newly diagnosed TB), G3 (TB without hepatotoxicity) and G4 (TB with hepatotoxicity). Adjusted *p*-values are presented using Kruskal–Wallis test followed by post hoc Dunn’s multiple comparison test (*p < 0.05, **p < 0.01, ***p < 0.001 and, ****p < 0.0001). Normal range: ALT = 2–41 U/L, AST = 2–40 U/L, ALP = 40–129 U/L, T.BIL. = 0–1.0 mg/dL, GGT = 5–61 U/L, Total protein = 6.8–8.3 (g/dL).

### Shotgun proteomic analysis and relative protein quantification

**Figure 1:**
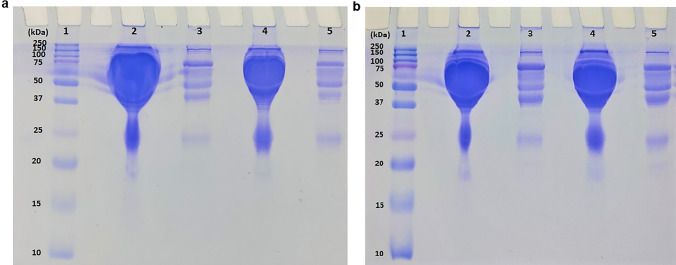
Representative images of SDS–PAGE: Protein differences of pooled serum samples are shown before and after depletion of two high abundant proteins including albumin and IgG (**a**) Groups 1 and 2 (Lane 1: Ladder, Lane 2: Pooled Group 1 serum, Lane 3: Two proteins depleted fraction of group 1, Lane 4: Pooled Group 2 serum and Lane 5: Two proteins depleted fraction of group 2) and (**b**) Groups 3 and 4 (Lane 1: Ladder, Lane 2: Pooled Group 3 serum, Lane 3: Two proteins depleted fraction of group 3, Lane 4: Pooled Group 4 serum and Lane 5: Two proteins depleted fraction of group 4.

After protein quantification in serum samples of defined study groups, 10 samples of each group were pooled taking equal protein concentration and two high abundant serum proteins (albumin and immunoglobulin G) were removed. SDS PAGE demonstrated efficient depletion of 90% of albumin (mol. wt. = 66.5 kDa) and immunoglobulin G (IgG) (mol. wt. = 150 kDa) which together constitute 50–70% and 10–25% of human serum proteins, respectively from pooled serum samples of defined study groups 1, 2, 3 and 4. The representative original gel images are shown in Fig. 1.

The depleted serum samples of each group were run in duplicates on LC–MS/MS. To avoid false protein identification, peptides with high confidence and proteins with a minimum of one unique peptide sequence were allowed for protein grouping. A total of 376 proteins with at least one unique peptide were identified, out of which 276 proteins were filtered out and relatively compared by applying a filter ‘found in samples’ has confidence ‘not found’ in at most 1 sample among defined study groups. By applying this filter, the proteins which were detected in at least 3 sample groups were considered for relative quantification and were further statistically and bioinformatically studied for differential expression and functional analysis.

### Comparative proteomic analysis and identification of signature proteins

**Figure 2:**
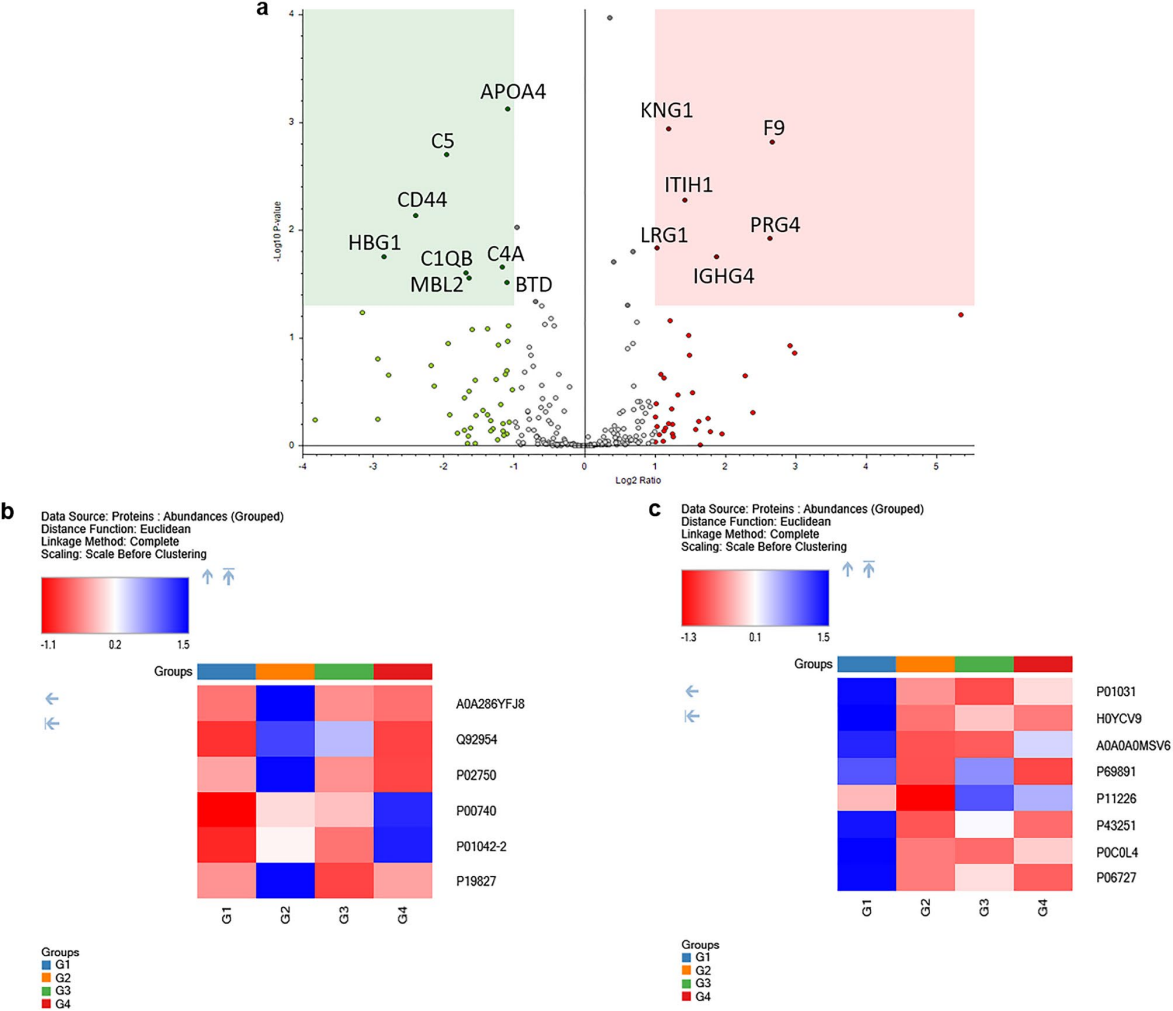
Volcano plot and heatmap representation of newly diagnosed TB (Group 2) versus healthy group (Group 1): Statistically significant normalized grouped abundance ratios of (**a**) Newly diagnosed TB versus a healthy group with − log10 *p*-value (Y) and log2 fold change (X). Heatmap of differentially expressed proteins from newly diagnosed TB versus healthy group comparison of (**b**) upregulated and (**c**) downregulated proteins where G1 (Healthy), G2 (newly diagnosed TB), G3 (TB without hepatotoxicity) and G4 (TB with hepatotoxicity) represent grouped abundance of each group. A *p*-value < 0.05 is considered as statistically significant. Heatmaps and volcano plot generated using Proteome Discoverer 2.5 (Thermo Scientific, Bremen, Germany).

Comparison of the newly diagnosed TB group (Group 2) versus the healthy group (Group 1) led to the identification of 14 significant differentially expressed proteins. Volcano plots highlighted a significant difference between the healthy and freshly diagnosed TB groups (Fig. 2a). Some of the upregulated proteins present in the serum of TB patients include Coagulation factor IX (F9), Proteoglycan 4 (PRG4), isoform LMW of Kininogen-1 (KNG1) and Leucine-rich alpha-2-glycoprotein (LRG1). In contrast, the proteins such as Apolipoprotein A-IV (APOA4), Mannose-binding protein C (MBL2) and CD44 antigen (Fragment) (CD44) were found to be downregulated in newly diagnosed TB patients (Group 2) as compared to healthy controls (Group 1) (Fig. 2b, c and Table 4).

**Table 4:** A comparison of significantly differentially expressed proteins from newly diagnosed TB (G2) versus healthy (G1) versus groups.

Accession	Description	Direction of change (G2 vs. G1)	#Unique Peptides	Gene Symbol	Abundance Ratio	*p*-value
P00740	Coagulation factor IX	↑	4	F9	6.4	0.002
Q92954	Proteoglycan 4	↑	6	PRG4	6.2	0.012
A0A286YFJ8	Immunoglobulin heavy constant gamma 4 (Fragment)	↑	6	IGHG4	3.7	0.018
P19827	Inter-alpha-trypsin inhibitor heavy chain H1	↑	12	ITIH1	2.7	0.005
P01042-2	Isoform LMW of Kininogen-1	↑	21	KNG1	2.3	0.001
P02750	Leucine-rich alpha-2-glycoprotein	↑	11	LRG1	2.0	0.015
P06727	Apolipoprotein A-IV	↓	32	APOA4	0.5	0.001
P43251	Biotinidase	↓	10	BTD	0.5	0.031
P0C0L4	Complement C4-A	↓	3	C4A	0.4	0.022
P11226	Mannose-binding protein C	↓	5	MBL2	0.3	0.028
A0A0A0MSV6	Complement C1q subcomponent subunit B (Fragment)	↓	5	C1QB	0.3	0.025
P01031	Complement C5	↓	28	C5	0.3	0.002
H0YCV9	CD44 antigen (Fragment)	↓	3	CD44	0.2	0.007
P69891	Hemoglobin subunit gamma-1	↓	2	HBG1	0.1	0.018

**Figure 3:**
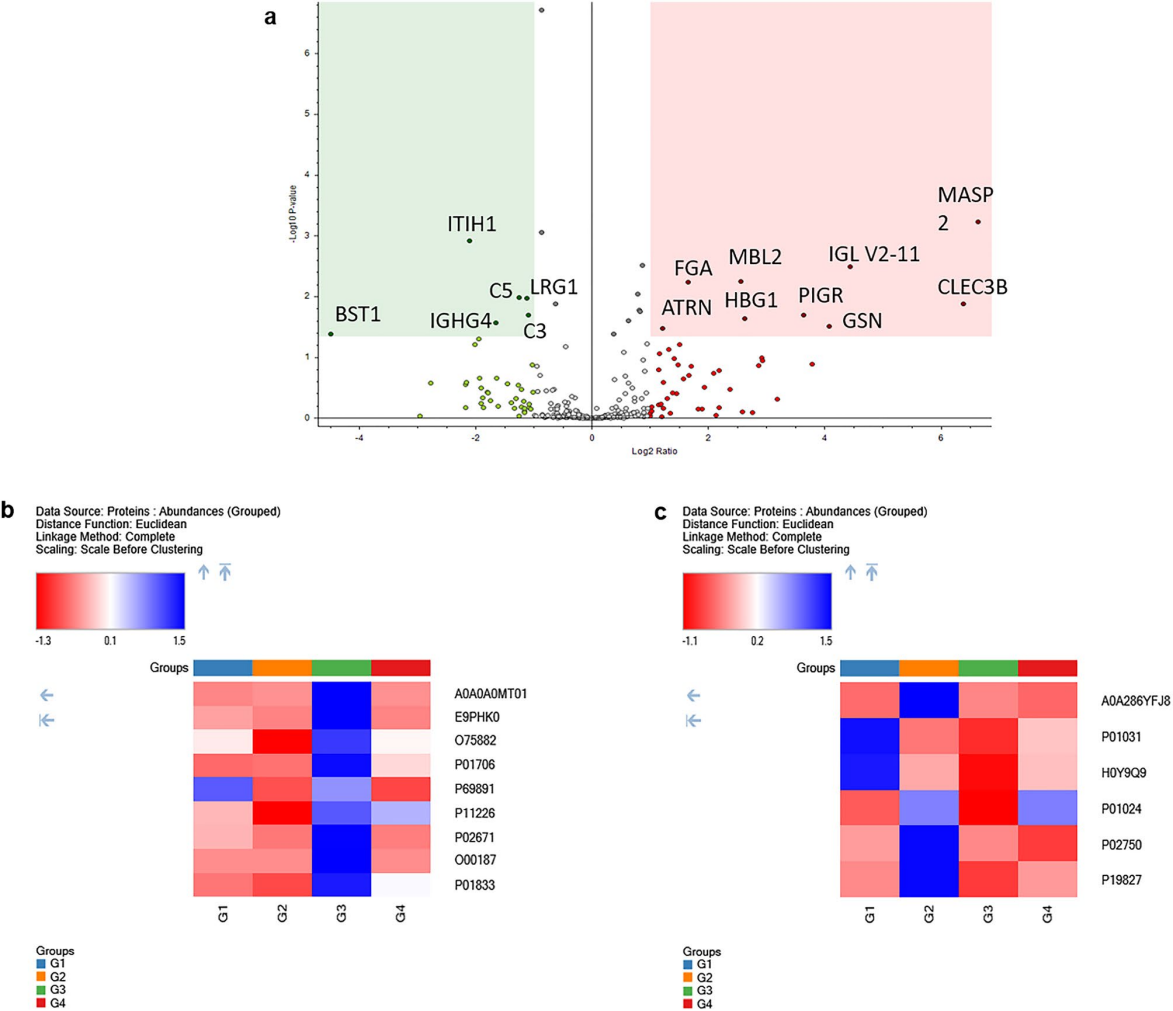
Volcano plot and heatmap representation of TB without hepatotoxicity (Group 3) versus newly diagnosed TB group (Group 2) groups: Statistically significant normalized grouped abundance ratios of (**a**) TB without hepatotoxicity versus newly diagnosed TB with − log10 *p*-value (Y) and log2 fold change (X). Heatmap of differentially expressed proteins from TB without hepatotoxicity versus newly diagnosed TB groups comparison of (**b**) upregulated and (**c**) downregulated proteins where G1 (Healthy), G2 (newly diagnosed TB), G3 (TB without hepatotoxicity) and G4 (TB with hepatotoxicity) represent grouped abundance of each group. A *p*-value < 0.05 is considered as statistically significant. Heatmaps and volcano plot generated using Proteome Discoverer 2.5 (Thermo Scientific, Bremen, Germany).

**Figure 4:**
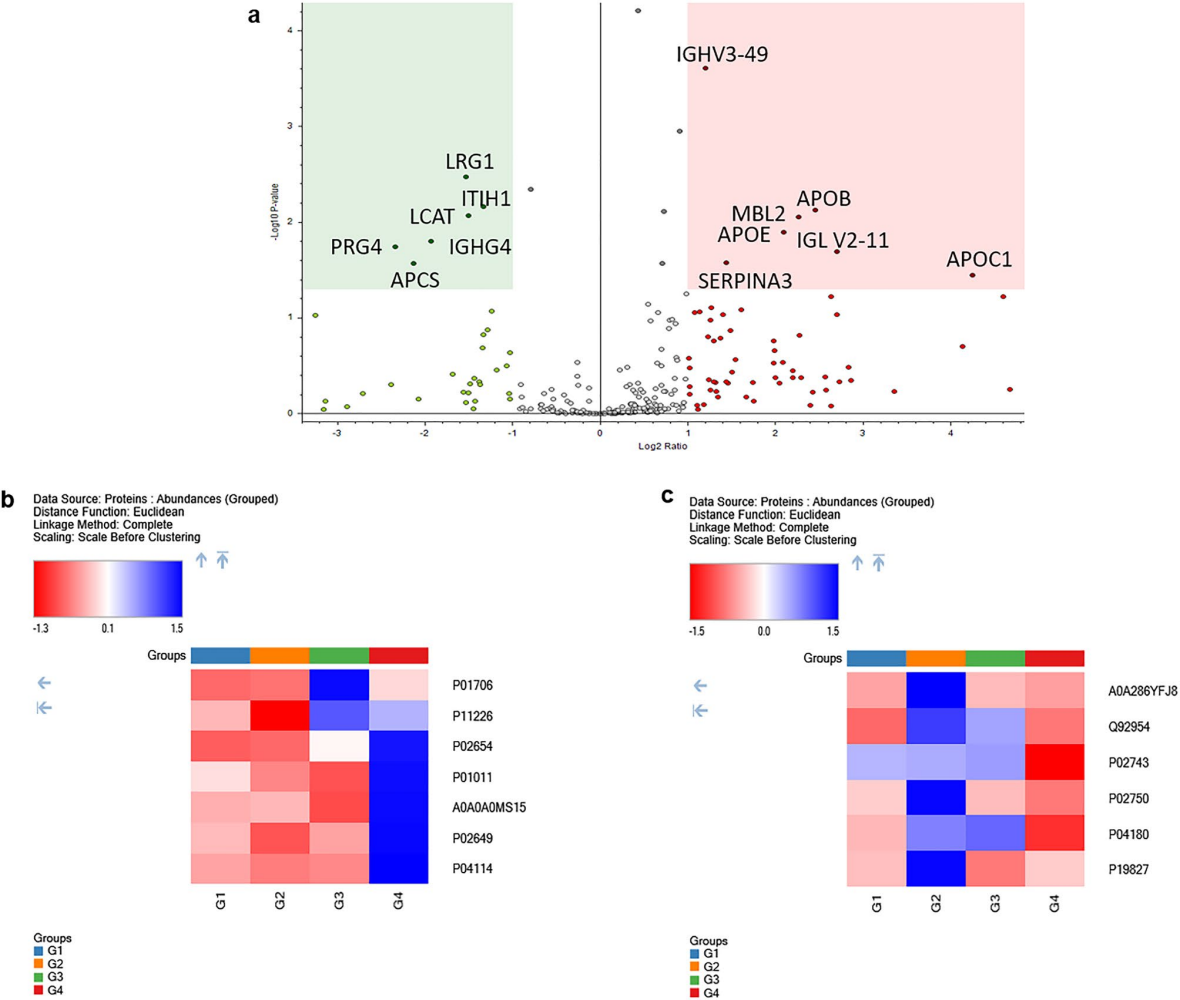
Volcano plot and heatmap representation of TB with hepatotoxicity (Group 4) versus newly diagnosed TB group (Group 2) groups: Statistically significant normalized grouped abundance ratios of (**a**) TB with hepatotoxicity versus newly diagnosed TB with − log10 *p*-value (Y) and log2 fold change (X). Heatmap of differentially expressed proteins from TB with hepatotoxicity versus newly diagnosed TB groups comparison of (**b**) upregulated and (**c**) downregulated proteins where G1 (Healthy), G2 (newly diagnosed TB), G3 (TB without hepatotoxicity) and G4 (TB with hepatotoxicity) represent grouped abundance of each group. A *p*-value < 0.05 is considered as statistically significant. Heatmaps and volcano plot generated using Proteome Discoverer 2.5 (Thermo Scientific, Bremen, Germany).

A comparison of TB without hepatotoxicity (Group 3) versus newly diagnosed TB group (Group 2) showed 15 significant differentially expressed proteins (Fig. 3a). Some of the upregulated proteins include Mannan-binding lectin serine protease 2 (MASP2), Tetranectin (CLEC3B), Mannose-binding protein C (MBL2), Complement C3 (C3), Complement C5 (C5) and Inter-alpha-trypsin inhibitor heavy chain H1 (ITIH1) (Table 5, Fig. 3b, c). Moreover, a comparison of TB with hepatotoxicity versus newly diagnosed TB groups revealed 13 differentially expressed proteins (Fig. 4a). The upregulated proteins include Apolipoprotein C-I (APOC1), Mannose-binding protein C (MBL2) and Apolipoprotein E (APOE) etc. In contrast, the observed downregulated proteins include LRG1, Serum amyloid P-component (APCS) and Proteoglycan 4 (PRG4) (Table 6, Fig. 4b, c).

**Table 5:** A comparison of significantly differentially expressed proteins from TB without hepatotoxicity (G3) versus newly diagnosed TB (G2) groups.

Accession	Description	Direction of change (G3 vs. G2)	# Unique peptides	Gene symbol	Abundance ratio	*p*-value
O00187	Mannan-binding lectin serine protease 2	↑	2	MASP2	100.0	0.001
E9PHK0	Tetranectin	↑	2	CLEC3B	84.0	0.013
P01706	Immunoglobulin lambda variable 2–11	↑	1	IGLV2-11	21.7	0.003
A0A0A0MT01	Actin-depolymerizing factor	↑	9	GSN	16.9	0.031
P01833	Polymeric immunoglobulin receptor	↑	11	PIGR	12.5	0.020
P69891	Hemoglobin subunit gamma-1	↑	2	HBG1	6.2	0.023
P11226	Mannose-binding protein C	↑	5	MBL2	5.9	0.006
P02671	Fibrinogen alpha chain	↑	6	FGA	3.1	0.006
O75882	Attractin	↑	24	ATRN	2.3	0.034
P01024	Complement C3	↓	107	C3	0.5	0.020
P02750	Leucine-rich alpha-2-glycoprotein	↓	11	LRG1	0.5	0.011
P01031	Complement C5	↓	28	C5	0.4	0.011
A0A286YFJ8	Immunoglobulin heavy constant gamma 4 (Fragment)	↓	6	IGHG4	0.3	0.027
P19827	Inter-alpha-trypsin inhibitor heavy chain H1	↓	12	ITIH1	0.2	0.001
H0Y9Q9	ADP-ribosyl cyclase/cyclic ADP-ribose hydrolase (Fragment)	↓	1	BST1	0.0	0.042

**Table 6:** The comparison of significantly differentially expressed proteins from TB with hepatotoxicity (G4) and newly diagnosed TB (G2) groups.

Accession	Description	Direction of change (G4 vs. G2)	# Unique peptides	Gene symbol	Abundance ratio	*p*-value
P02654	Apolipoprotein C-I	↑	3	APOC1	19.1	0.036
P01706	Immunoglobulin lambda variable 2–11	↑	1	IGLV2-11	6.5	0.020
P04114	Apolipoprotein B-100	↑	211	APOB	5.5	0.008
P11226	Mannose-binding protein C	↑	5	MBL2	4.8	0.009
P02649	Apolipoprotein E	↑	14	APOE	4.3	0.013
P01011	Alpha-1-antichymotrypsin	↑	16	SERPINA3	2.7	0.027
A0A0A0MS15	Immunoglobulin heavy variable 3–49	↑	2	IGHV3-49	2.3	0.000
P19827	Inter-alpha-trypsin inhibitor heavy chain H1	↓	12	ITIH1	0.4	0.007
P04180	Phosphatidylcholine-sterol acyltransferase	↓	8	LCAT	0.4	0.009
P02750	Leucine-rich alpha-2-glycoprotein	↓	11	LRG1	0.3	0.003
A0A286YFJ8	Immunoglobulin heavy constant gamma 4 (Fragment)	↓	6	IGHG4	0.3	0.016
P02743	Serum amyloid P-component	↓	7	APCS	0.2	0.027
Q92954	Proteoglycan 4	↓	6	PRG4	0.2	0.018

**Figure 5:**
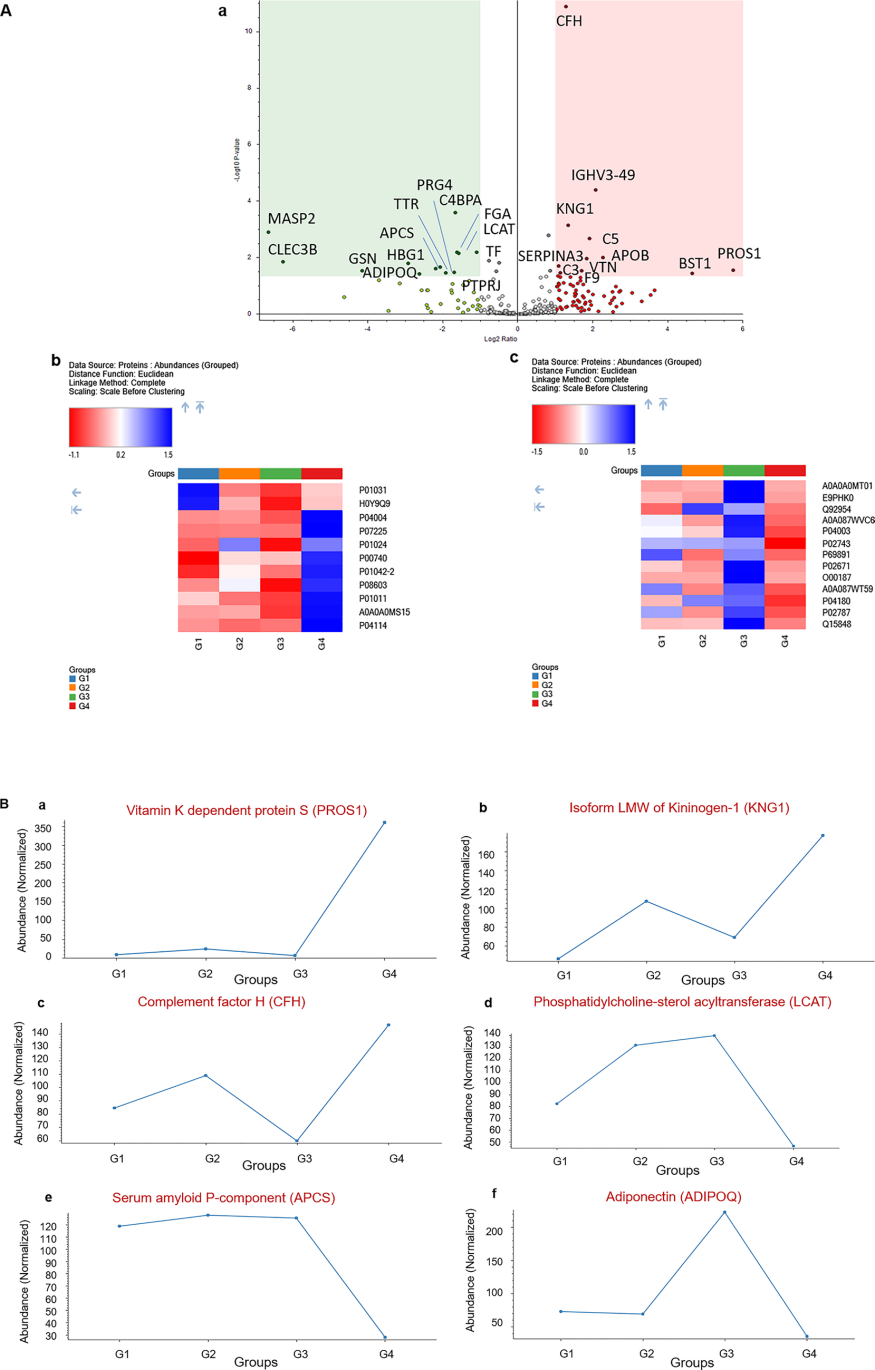
Volcano plot and heatmap representation of TB with hepatotoxicity (Group 4) versus TB without hepatotoxicity (Group 3) groups: Statistically significant normalized grouped abundance ratios of (**A,a**) TB with hepatotoxicity versus TB without hepatotoxicity with − log10 *p*-value (Y) and log2 fold change (X). Heatmap of differentially expressed proteins from TB with hepatotoxicity versus TB without hepatotoxicity groups comparison of (**A,b**) upregulated and (**A,c**) downregulated proteins. (**B**) The trend chart of signature proteins including (a) PROS1, (b) KNG1, (c) CFH, (d) LCAT, (e) APCS and (f) ADIPOQ which showed the potential to distinguish the hepatotoxicity group from other groups where G1 (Healthy), G2 (newly diagnosed TB), G3 (TB without hepatotoxicity) and G4 (TB with hepatotoxicity) represent grouped abundance of each group. A *p*-value < 0.05 is considered as statistically significant. Heatmaps and volcano plot generated using Proteome Discoverer 2.5 (Thermo Scientific, Bremen, Germany).

Furthermore, TB with hepatotoxicity group (Group 4) in comparison to TB without hepatotoxicity (Group 3) showed 24 significant differentially expressed proteins (Table 7, Fig. 5A a–c). Vitamin K-dependent protein S (PROS), ADP-ribosyl cyclase/cyclic ADP-ribose hydrolase (Fragment) (BST1) and Apolipoprotein B-100 (APOB) were found to be upregulated whereas downregulated proteins include Phosphatidylcholine-sterol acyltransferase (LCAT), Adiponectin (ADIPOQ), Serum amyloid P-component (APCS), Proteoglycan 4 (PRG4) and Transthyretin (TTR). Some of the observed proteins were similar to the identified proteins in TB with hepatotoxicity (Group 4) compared to newly diagnosed TB group (Group 2) (Fig. 4a) or with the healthy group (Group 1) (data shown in supplementary Fig. 1). Noteworthy, some protein signatures like PROS1, KNG1, CFH, LCAT, APCS and ADIPOQ showed the potential to distinguish the hepatotoxicity group from other defined study groups. However, only APCS protein showed significant downregulation in Group 4 as compared to Groups 1,2,3 suggesting its potential role in anti-tubercular drug induced hepatotoxicity (Fig. 5B).

**Table 7:** The comparison of significantly differentially expressed proteins from TB with hepatotoxicity (G4) versus TB without hepatotoxicity (G3) groups.

Accession	Description	Direction of change (G4 vs. G3)	# Unique peptides	Gene symbol	Abundance ratio	*p*-value
P07225	Vitamin K-dependent protein S	↑	19	PROS1	54.1	0.029
H0Y9Q9	ADP-ribosyl cyclase/cyclic ADP-ribose hydrolase (Fragment)	↑	1	BST1	25.3	0.037
P04114	Apolipoprotein B-100	↑	211	APOB	4.8	0.010
A0A0A0MS15	Immunoglobulin heavy variable 3–49	↑	2	IGHV3-49	4.2	0.000
P01031	Complement C5	↑	28	C5	3.8	0.002
P01011	Alpha-1-antichymotrypsin	↑	16	SERPINA3	3.6	0.011
P04004	Vitronectin	↑	9	VTN	3.3	0.030
P01042-2	Isoform LMW of Kininogen-1	↑	21	KNG1	2.6	0.001
P08603	Complement factor H	↑	28	CFH	2.5	0.000
P00740	Coagulation factor IX	↑	4	F9	2.2	0.035
P01024	Complement C3	↑	107	C3	2.1	0.020
P02787	Serotransferrin	↓	53	TF	0.5	0.007
P04180	Phosphatidylcholine-sterol acyltransferase	↓	8	LCAT	0.3	0.007
P02671	Fibrinogen alpha chain	↓	6	FGA	0.3	0.006
P04003	C4b-binding protein alpha chain	↓	25	C4BPA	0.3	0.000
A0A087WVC6	Protein-tyrosine-phosphatase	↓	2	PTPRJ	0.3	0.033
Q92954	Proteoglycan 4	↓	6	PRG4	0.3	0.036
A0A087WT59	Transthyretin	↓	12	TTR	0.2	0.022
P02743	Serum amyloid P-component	↓	7	APCS	0.2	0.025
Q15848	Adiponectin	↓	2	ADIPOQ	0.2	0.039
P69891	Hemoglobin subunit gamma-1	↓	2	HBG1	0.1	0.016
A0A0A0MT01	Actin-depolymerizing factor	↓	9	GSN	0.1	0.029
E9PHK0	Tetranectin	↓	2	CLEC3B	0.0	0.014
O00187	Mannan-binding lectin serine protease 2	↓	2	MASP2	0.0	0.001

### GO annotation and pathway analysis of differentially expressed proteins

**Figure 6:**
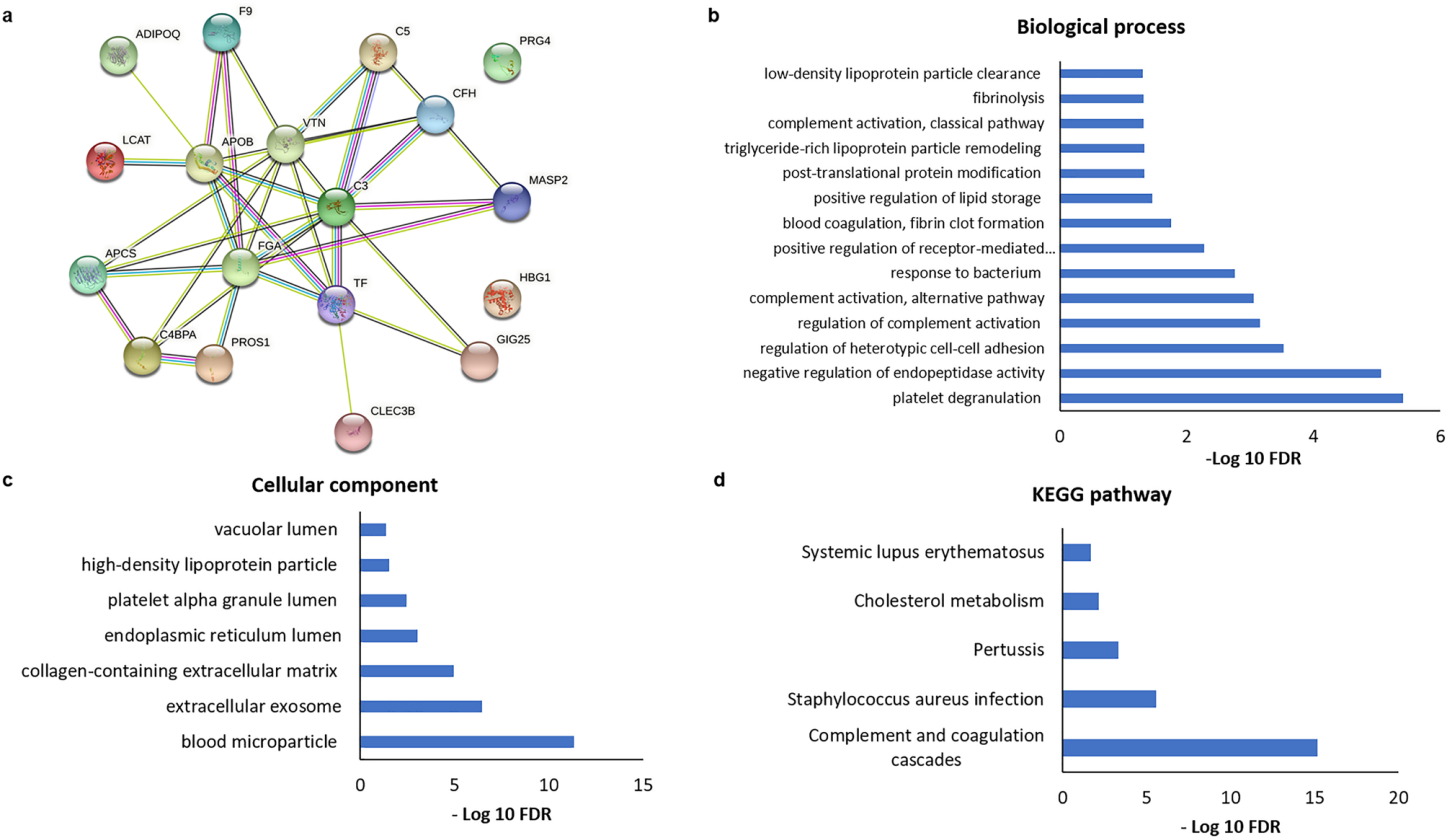
Bioinformatics analysis of differentially expressed proteins: A strong interactome network (**a**) of modulated proteins by String 11.0 among TB with hepatotoxicity and TB without hepatotoxicity group comparison^42^. Functional enrichment analysis utilizing Gene Ontology^43^ (**b** and **c**) shows that most modulated proteins are involved in complement activation (alternative pathway), triglyceride-rich lipoprotein particle remodelling, fibrinolysis with platelet degranulation, and belongs to high-density lipoprotein particle, blood microparticle and platelet alpha granule lumen of the cell. (**d**) KEGG pathway enrichment analysis^44^ shows that modulated proteins are primarily engaged in complement and coagulation cascades and cholesterol metabolism.

The gene symbol of differentially expressed proteins identified among TB with hepatotoxicity versus TB without hepatotoxicity groups was listed in String 11.0 for interaction analysis. It showed a strong interaction network (Fig. 6a). The gene ontology (GO) functional enrichment analysis by biological process and cellular component was performed with the differentially expressed proteins (Fig. 6b, c). It showed that proteins were mostly involved in biological processes like complement activation, alternative pathway (CFH, C5 and C3), triglyceride-rich lipoprotein particle remodelling (LCAT and APOB), fibrinolysis (PROS1 and FGA) with platelet degranulation (CLEC3B, PROS1, FGA, TF and SERPINA3) and were part of high-density lipoprotein particle (APOB and LCAT), blood microparticle (PROS1, GSN, CFH, C3, APCS, C4BPA, VTN, FGA, TF and SERPINA3) and platelet alpha granule lumen (PROS1, FGA and SERPINA3) of the cell. Thus, the modulated proteins were involved in complement and coagulation cascades (F9, C5, VTN, C3, FGA, C4BPA, CFH, PROS1, MASP2) and cholesterol metabolism (APOB, LCAT), which might play a role in drug-induced liver injury (Fig. 6d).

### Validation of serum amyloid P component protein (APCS) in serum samples of different study groups

**Figure 7:**
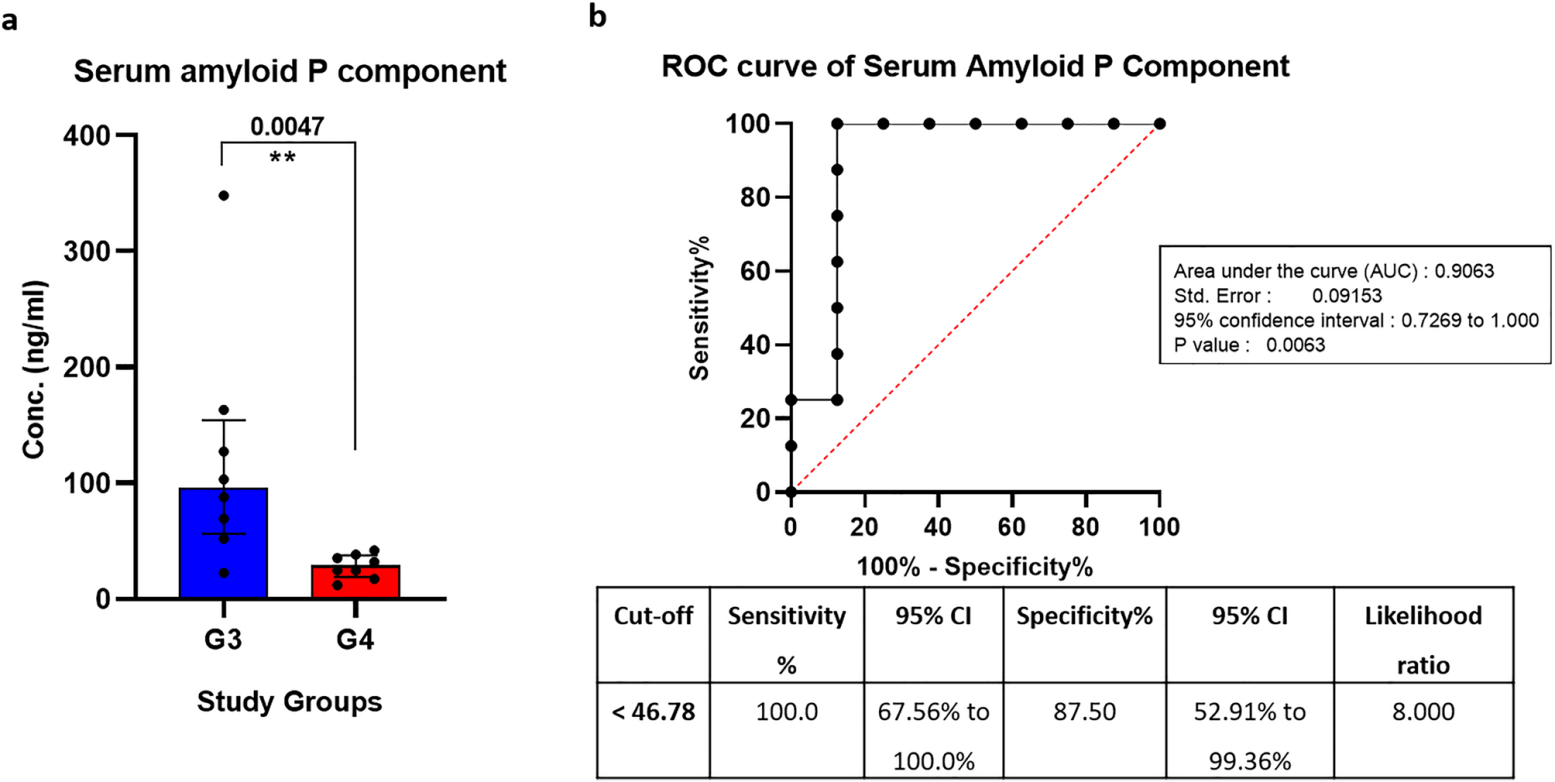
Boxplot representation and ROC curve analysis of APCS protein: The levels of significantly downregulated (**a**) serum amyloid P component (APCS) in serum samples of G4 (TB with hepatotoxicity) versus G3 (TB without hepatotoxicity) study subjects. Mann–Whitney test was used to compare serum APCS protein levels. (**b**) ROC and Youden index analysis was done for the APCS to investigate their discriminatory potential between TB patients with and without hepatotoxicity (n = 8 for each group; Conc. = concentration; CI: Confidence interval).

ELISA was used for validation of APCS protein which was observed to be downregulated specifically in TB with hepatotoxicity group (Group 4) in comparison to TB without hepatotoxicity (Group 3). APCS protein showed a significant downward trend and statistical analysis revealed comparable results to those obtained in discovery phase (Fig. 7a). Receiver Operating Characteristic (ROC) curve analysis showed 90% AUC (area under the curve) values with 100% sensitivity and 87% specificity as revealed by Youden index analysis, confirming its diagnostic potential that can further be explored in larger cohort (Fig. 7b).

## Discussion

Clinically, an attractive diagnostic tool for characterizing DILI is based on measuring biochemical markers including ALT, AST, ALP, Total Bilirubin and GGT in serum^16,17^. Despite the availability of these traditional biomarkers, the investigations to find a suitable candidate(s) that can predict DILI at an earlier onset are still in progress as conventional markers lack sensitivity and specificity, which further possess limitations in deciding subsequent treatment regimens. Proteomics, along with other omics technologies, has been explored to find out extensive list of proteins, microRNAs and many other biomarkers related to DILI but still no biomarker is in clinical utility^15^. In present study, 376 proteins were quantified by label-free quantification in pooled serum samples and the number of quantified proteins is concordant with other proteomics-based studies in serum of TB patients, while because of technical variations, a higher number of proteins were also identified in other studies^18–20^. Prior to mass spectrometry run, the serum samples were depleted of albumin and IgG as the high abundant proteins depletion is one of the common strategies to identify low abundant proteins which holds biomarker potential^21,22^.

Out of identified 376 proteins, 276 proteins were filtered out and compared among defined study groups. Newly diagnosed TB group versus healthy group comparison presented many upregulated proteins, for instance, F9, PRG4, KNG1 and LRG1. While the proteins such as APOA4, BTD, MBL2, C4A, C5, HBG1 and others were found to be significantly decreased. The findings are consistent with the recent available literature on serum proteomic profiling of healthy and TB patients^18,23,24^. The levels of Mannose binding lectin-2 (MBL2) were found to be decreased in TB patients versus the healthy group. In contrast, an increasement in its level was observed upon initiation of anti-TB drug treatment, which remained high in the toxic group, suggesting its involvement in the pathogenesis of TB. MBL is known to play a potential role in innate immunity and is decreased in TB patients, further, increasing susceptibility to infections^25^.

Next, to evaluate the effect of anti-TB treatment on the identified protein profile, the newly diagnosed TB group was compared with subjects with hepatotoxicity and without hepatotoxicity groups. Interestingly, the proteins including IGHG4, ITIH1, LRG1, MBL2 and LRG1 showed an opposite trend of differential expression when the newly diagnosed TB group was compared among TB group without hepatotoxicity and healthy group, respectively, suggesting an influence of anti-TB drug treatment. Other proteins, including CLEC3B, PIGR, FGA and ATRN (upregulated proteins) and C3, C5 and BST1 (downregulated proteins), were also differentially expressed, suggesting the possible effect of anti-TB drugs on the expression of enlisted proteins. The observed experimental findings are similar to the previously reported studies which suggest that reduced C3 levels are correlated with the improved treatment outcome in TB patients^26,27^.

Furthermore, as the study primarily focused on characterizing the protein signatures significantly modulated in TB patients with hepatotoxicity, a subset of proteins were observed to be differentially expressed among TB with hepatotoxicity versus those without hepatotoxicity groups. Some specific proteins include PROS1, APOB, IGHV3-49, SERPINA3, VTN, KNG1, CFH, F9 (upregulated) and TF, LCAT, FGA, C4BPA, PTPRJ, TTR, APCS, ADIPOQ, GSN, CLEC3B, MASP2 (downregulated). Some of the observed proteins, including SERPINA3, KNG1 and APCS, are involved in TB pathogenesis^28–30^. The Complement C3 (C3) protein, along with Complement factor H (CFH), was found to be upregulated in TB with the hepatotoxicity group. CFH is one of the primary regulators of the alternative pathway. Ultimately, all the three complement pathways (including classical, lectin and alternative) lead to activation of C3 convertase and downstream cleavage of C3. This leads to opsonization of bacteria by macrophages, and activation of C5 convertase which may result in mycobacterial cell lysis by forming a membrane attack complex^31^. The increased levels of C3 as observed in the hepatotoxicity group might be linked with TB treatment failure and reduced C3 levels have been correlated with the improved treatment outcome^26^. Our findings are supported by available literature where increased oxidative stress is linked with complement-dependent (particularly C3, CFH and C5 components) inflammatory responses^32,33^. Vitamin K-dependent protein S (PROS1) levels were also upregulated in the hepatotoxicity group. It is known that PROS1 is synthesized in the liver which plays a central role in blood coagulation and acts as an anticoagulant plasma protein^34^. A decrease in Adiponectin (ADIPOQ) levels, an adipokine, was found in pulmonary TB patients. At the same time, an increase was observed after initiation of antitubercular therapy^35^. However, surprisingly in our study, we observed that the levels of Adiponectin showed a downward trend in the hepatotoxicity group suggesting further suggesting its diagnostic potential in hepatotoxicity group. Another protein i.e., serum amyloid P-component (APCS) showed a similar trend in all the three study groups (Group 1, 2 and 3) except in the TB with hepatotoxicity group (Group 4), where its level showed a significant decrease. Serum amyloid P-component (APCS gene) along with CRP are classic pentraxin proteins. APCS protein plays a crucial role in regulating the matrix formation and provides resistance to infectious agents including *mycobacteria*^36–38^. One targeted proteomics study using extracellular vesicles highlights that levels of APCS and pro-platelet basic protein were significantly decreased in liver fibrosis progression, patients with liver cirrhosis, acute hepatitis and non-alcoholic fatty liver disease patients^39^. However, no reports are avaibale where levels of APCS have been correlated with anti-tubercular hepatotoxicity. Based on its specific and significant decrease in hepatotoxicity group and being a critical protein in combating the infection, APCS protein was shortlisted for further validation by ELISA. The validation phase data correlated well with the discovery phase suggesting its biomarker potential in differentiating anti-tubercular drug-induced liver injury from other defined study groups. Henceforth, our resuts suggest APSC as a potential candidate protein to further explore its role in anti-tubercular drug induced liver injury. This study can further be strengthened by validating other essential signature proteins (PROS1, KNG1, CFH, LCAT and ADIPOQ) in larger cohort which have shown significant differential expression in the TB group with hepatotoxicity as compared to without hepatotoxicity group. Also, the present study is a cross-sectional study that can be redesigned to a randomized one to diagnose the transition of markers in different stages of the disease.

## Conclusion

Serum proteomic analysis revealed significant changes in the expression profile of signature proteins in TB with hepatotoxicity patients versus TB without hepatotoxicity patients. An interplay among differentially regulated proteins highlighted their involvement in complement and coagulation cascades and cholesterol metabolism, which might play a significant role in AT-DILI in TB patients. Among differentially expressed proteins including PROS1, KNG1, CFH (upregulated) and APIPOQ, APCS and LCAT (downregulated), APCS was shortlisted for validation based on functional relevance with hepatotoxicity. The protein showed high discriminative ability with 100% sensitivity and 87% specificity. Henceforth, our results suggest APCS as a potential candidate protein which further can be explored to study its potential role in anti-tubercular drug induced liver injury.

## Methods

Protein quantification was performed by Pierce™ BCA Protein Assay Kit (Thermo Fisher Scientific, USA), and high abundant protein depletion was achieved by Aurum™ Serum Protein Mini Kit (Bio-Rad). Trypsin (Promega gold standard), Guanidine Hydrochloride (GnHCl), Dithiothreitol (DTT), iodoacetic acid (IAA), and trifluoroacetic acid (TFA) were procured from Sigma-Aldrich while LC–MS Grade water and Pierce™ C18 Spin columns (Cat. 89870) were procured from Thermo Fisher Scientific Inc, USA. Protein ladder 10–250 kDa (Precision Plus Protein™ Dual Color Standards) was purchased from BioRad.

### Study subjects

The Institute Ethics Committee (IEC) of Postgraduate Institute of Medical Education and Research (PGIMER), Chandigarh reviewed and approved the study protocol with ref. no. IEC No. 10/2014-72 and NK/5864/MSc/137 and all procedures were performed as per *Declaration of Helsinki* guidelines. Study subjects were recruited, and blood samples were withdrawn from patients attending medical OPD and DOTS Center, PGIMER, Chandigarh, considering the inclusion and exclusion criteria of the study. Briefly, patients of either sex with any type of TB in the age range of 15–65 years were enrolled. The subjects were divided into four groups i.e., Healthy control subjects having no signs and symptoms of TB and were free from any liver disease (Group 1), newly diagnosed TB subjects not on TB treatment (Group 2), TB patients on antitubercular drug therapy not having hepatotoxicity (Group 3), and TB patients on anti-TB treatment and having hepatotoxicity (Group 4) (ALT or AST ≥ three-fold rise). Groups 3 and 4 received the same anti-TB drugs (essentially with RIF, PZA and INH);. Along with biochemical investigations, chest X-ray, sputum test for AFB positivity, Montoux test or microscopic examination for TB confirmation was assessed by concerned physician attending chest or medical clinic. Height and weight were also measured for BMI calculation using formula: weight (kg)/height (m^2^). Patients with human immunodeficiency virus infection, viral hepatitis, simultaneous consumption of other potentially hepatotoxic medications like methotrexate, phenytoin, fluconazole and valproate etc., chronic liver disease, long-term alcoholics and pregnancy were excluded^40^.

### Blood sample collection and categorization of study subjects

Serum was separated from the collected blood samples by centrifugation at 1500 g × 10 min and kept at − 80 °C until further analysis. Biochemical parameters including ALT, AST, ALP, TBIL, GGT and total protein levels were estimated in serum of recruited study subjects on Roche/Hitachi Cobas c-8000 system. Values ≥ three times the upper limit of normal (ULN) of ALT, AST and TBIL were considered as criteria of hepatotoxicity^41^. After screening, 40 patients were included, categorized, and distributed equally in defined study groups. The demographic data of recruited patients in the respective study groups are summarized in Table 2. The individual serum samples of each representative group were pooled by considering that an equal amount of protein was taken from each sample. The protein concentration was measured by by Nanodrop One Microvolume UV–Vis spectrophotometer (Thermo Scientific™) and by BCA assay using bovine serum albumin as standard. Serum dilutions (1:100) were prepared using distilled water.

### Depletion of high abundant proteins and top–down assessment by SDS-PAGE

Two highly abundant proteins (albumin and immunoglobulin G) were removed using a serum-aurum kit as per the manufacturer’s protocol. Briefly, 60 µl of each pooled serum sample was diluted with 180 µl of Aurum serum protein binding buffer and was placed over the resin bed of the column. The column was vortexed and incubated for 15 min., centrifuged at 10,000 × g for 20 s, and depleted fractions were collected. The protein concentration thus obtained from depleted fractions was quantified by BCA assay. For a gross assessment of efficient depletion, 10 µg protein /gel lane was separated on 12% SDS PAGE, followed by coomassie blue staining to visualize protein bands.

### In-solution digestion

For LC–MS/MS analysis, 20–25 µg of the depleted protein samples suspended in the binding buffer of the serum-aurum kit was acetone precipitated (3 h at − 20 °C) and centrifuged at 22,000 × g at 4 °C for 10 min. The obtained pellet was air-dried and re-suspended in hot (90 °C) 6 M guanidine hydrochloride (GnHCl)/0.1 M Tris solution. Freshly prepared 10 mM DTT was added as a reducing agent followed by incubation at 65 °C for 45 min. in the dark. Then, 50 mM freshly prepared IAA was added as an alkylating agent and incubated in the dark for 45 min. Samples were diluted with LC–MS grade water to achieve 0.6 M GnHCl concentration, and pH was maintained between 8 and 8.5. The protein mixture was digested with trypsin at an enzyme-to-protein ratio of 1:20 (w/w) to generate peptides and kept at 37 °C for 16 h. Trypsin activity was quenched by adding 10% TFA (2–3 µl), and pH was maintained at 2. The peptide samples were purified and concentrated using the Pierce^®^ C18 Spin columns as per the manufacturer’s instructions. After clean-up, the peptide mixture was quantified by measuring absorption at 280 nm using Nanodrop.

### LC–MS/MS analysis

Depleted serum fractions of each group were run in duplicates on Orbitrap Fusion Tribrid Mass Spectrometer (Thermo Fisher Scientific). Peptide samples (~ 1ug) were loaded on reverse phase EASY-Spray Acclaim PepMap C18 Column (15 cm × 75 μm I.D., packed with C18 resin, three μm particle size, 100 Å; Thermo Scientific, Part No ES800), coupled to Easy NanoLC 1200 system (Thermo Fisher Scientific, San Jose, US). The peptide separation was achieved with a constant flow rate of 300 nl/min using a linear gradient of 5–95% solvent B (80% ACN in 0.1% formic acid) with solvent A (2% ACN in 0.1% formic acid) for 95–100 min. MS master scan was acquired at Orbitrap mass analyzer in the m/z range of 375–1600 with a mass resolution of 120,000 at m/z 200. Data-dependent MS/MS acquisition was achieved by IonTrap mass analyzer with 3s as cycle time. Quadrupole with an isolation window of 1.2 m/z was selected as an isolation mode. AGC target was set to 5000, and the maximum injection time was 45 ms. The precursor ion fragmentation with charge states 2–5 was achieved using higher-energy collisional dissociation at 30% collision energy. The lock mass option was enabled for real-time calibration using polycyclodimethylsiloxane (m/z, 445.12) ions.

### Data analysis

The raw files generated for each sample were analyzed by Proteome Discoverer 2.5 (Thermo Scientific, Bremen, Germany) for protein identification. Database searches were carried out using Sequest HT algorithms with percolator (Strict FDR = 0.01 and relaxed FDR = 0.05) against UniProt Human database (ID: UP000005640, 96,807 sequences, last modified on 3/9/2020). The precursor mass range of 350–5000 Da and S/N threshold was set as 1.5 for generating the peak list file. The parameters set for database searches include trypsin as a protease with two missed cleavages and a minimum peptide length of 6 aa, Oxidation/+15.995 Da (M) as dynamic oxidation and Carbamidomethyl/+57.021 Da (C) as static Modification. Precursor Mass Tolerance of 10 ppm and fragment Mass Tolerance of 0.6 Da were allowed. Calculation based on the score was used as peptide validator with target FDR (False discovery rate) for Peptide-Spectrum Matches (PSMs) as 1% (Strict) and 5% (Relaxed), and similar target FDR criteria were set for peptides as general validation settings. Peptide and protein filters were set so that peptides with at least high confidence and proteins with a minimum of one peptide sequence were allowed, and the strict parsimony principle was true for protein grouping. Peptides selected for quantification were Unique + Razor. Precursor quantification was based on precursor intensity which was used as precursor abundance value. Generated data was normalized with normalization mode selected as the total peptide amount. Protein abundance calculation was based on summed abundances.

### Statistical and bioinformatic analysis

For differential expression, the relative abundance ratio of ≥ two-fold was used for up-regulation and ≤ 0.5-fold abundance ratio for down-regulation, where protein ratio calculation was protein abundance-based. *p*-value was calculated by applying ANOVA (Individual proteins) with Tukey HSD posthoc analysis as a hypothetical test, and a value < 0.05 was considered a significant abundance ratio. The volcano plots and heatmaps of differentially expressed proteins were exported from Proteome Discoverer 2.5 (Thermo Scientific, Bremen, Germany). The statistically significant differentially expressed proteins were analyzed by STRING 11.0 for visualization of interactome among the proteins^42^. Moreover, the protein–protein interactions was followed by STRING in-built Gene Ontology (including biological processes and cellular components) and KEGG pathway for functional annotation and enrichment analysis^43,44^. For Gene ontology analysis, fisher’s exact test was selected to calculate the *p*-value with a cutoff of < 0.001 as statistical criteria. FDR was calculated as test correction for functional enrichment, and values were represented as − log10 FDR. While for KEGG pathway anaylsis, FDR was calculated according to Benjamini and Hochberg’s test.

### ELISA of serum amyloid P component protein and receiver operating characteristic analysis

The shortlisted Serum amyloid P component (APCS) protein from TB with hepatotoxicity versus without hepatotoxicity group were chosen for validation using ELISA kit (Elabscience): E-EL-H1279. Briefly, the collected serum samples (8 TB patients with hepatotoxicity and without hepatotoxicity groups, respectively) were diluted in the sample diluent provided with the kit following the manufacturer’s instructions and immunodetection was achieved. The absorbance was read at 450 nm in a Tecan—Microplate Reader—Infinite 200 Pro (DKSH Holding Ltd.). The diagnostic accuracy of APCS protein was evaluated by the receiver operator characteristics (ROC) curve analysis. The cut-off values were evaluated by calculating the Youden Index in order to maximize the sensitivity and specificity. Statistical analysis (Mann–Whitney) for ELISA was performed using GraphPad Prism 8.0.2.

### Ethics approval

The institutional ethical committee (IEC) approved the study with ref No. (IEC No. 10/2014-72 and NK/5864/MSc/137.

### Consent to participate

Informed consent was obtained from all the participants included in the study.

## Supplementary Information

Supplementary Figure 1.

## Data Availability

The data generated or analyzed has been included in the manuscript. The raw files has been submitted in MassIVE repository with ID: MSV000088373 and is publicly available.
