# Supplementary material for: Proteomic-based identification of APCS as candidate protein for diagnosis of patients exhibiting anti-tubercular drug induced liver injury
Source: Sci Rep. 2023 Jun 22;13:10135. doi: 10.1038/s41598-023-35930-x (PMC10287637; doi:10.1038/s41598-023-35930-x)
Supplement: Supplementary file 1 — Supplementary Figure 1. [file 41598_2023_35930_MOESM1_ESM.pdf]

Supplementary Figures

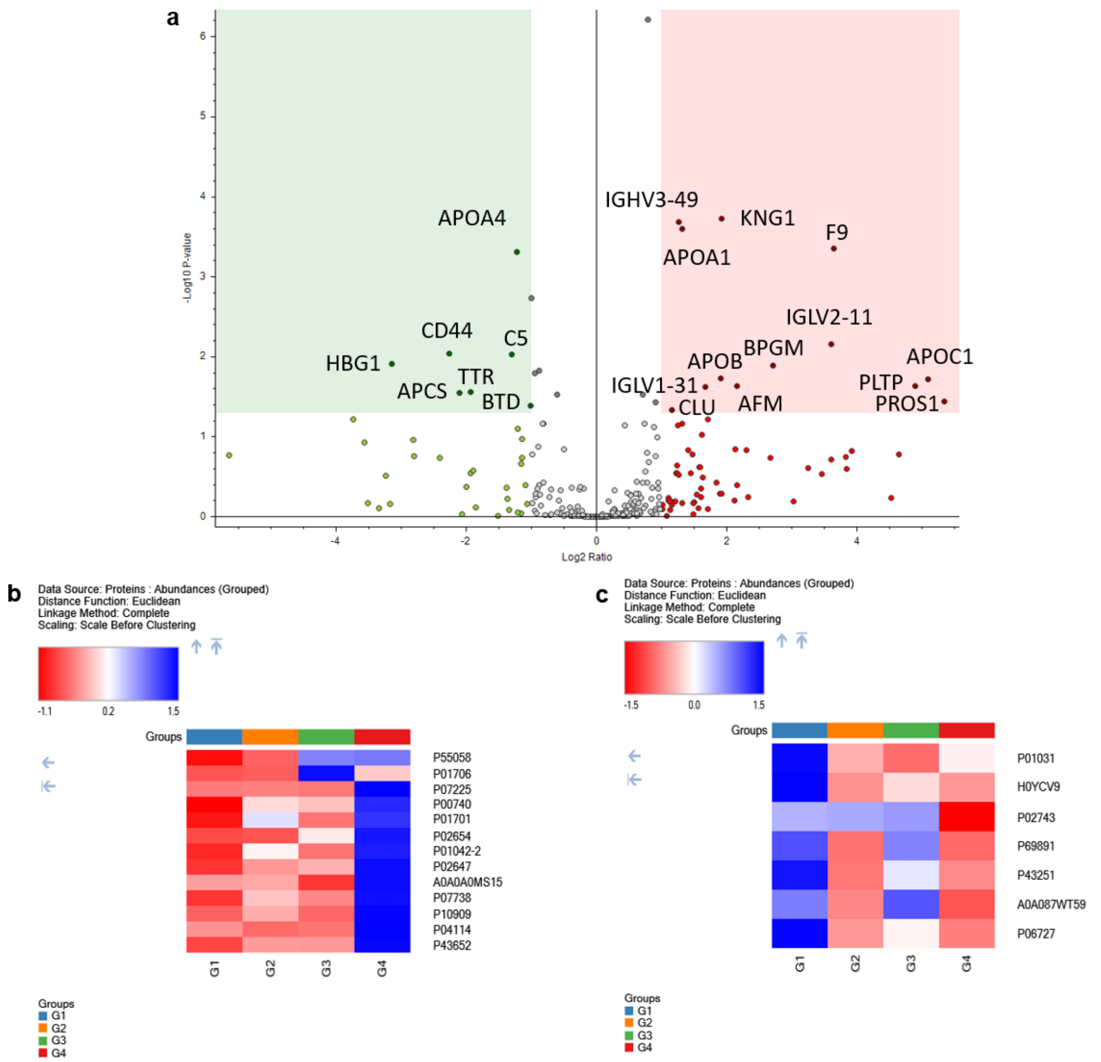

Supplementary Fig. 1: Volcano plot representation of statistically significant abundance ratios of (A) TB with hepatoxicity versus healthy group with -log10 p-value (Y) and log2 fold change (X). Heatmap of differentially expressed proteins of TB with hepatoxicity versus healthy groups comparison of (B) upregulated and (C) downregulated proteins where G1 (Healthy), G2 (newly diagnosed TB), G3 (TB without hepatoxicity) and G4 (TB with hepatoxicity) represent normalized grouped abundance of each group. A p-value < 0.05 is considered as statistically significant. Heatmaps and volcano plot generated using Proteome Discoverer 2.5 (Thermo Scientific, Bremen, Germany).
